# Familial clustering of diabetic kidney disease and retinopathy in offspring of parents with type 1 diabetes: a population-based study

**DOI:** 10.1016/j.eclinm.2025.103232

**Published:** 2025-05-06

**Authors:** Isabella Herrlin, Fanny Jansson Sigfrids, Niina Sandholm, Lena M. Thorn, Per-Henrik Groop, Valma Harjutsalo

**Affiliations:** a Folkhälsan Research Center, Biomedicum Helsinki, Helsinki, Finland; b Department of Nephrology, University of Helsinki and Helsinki University Hospital, Helsinki, Finland; c Research Program for Clinical and Molecular Metabolism, Faculty of Medicine, University of Helsinki, Helsinki, Finland; d Department of General Practice and Primary Health Care, University of Helsinki and Helsinki University Hospital, Helsinki, Finland; e Department of Diabetes, Central Clinical School, Monash University, Melbourne, VIC, Australia; f Baker Heart and Diabetes Institute, Melbourne, VIC, Australia

**Keywords:** Albuminuria, Diabetic nephropathy, Diabetic retinopathy, Epidemiology, Type 1 diabetes

## Abstract

**Background:**

Familial clustering of diabetic kidney disease and diabetic retinopathy has been reported among siblings with type 1 diabetes. We aimed to further unveil the phenomenon among parent-offspring pairs.

**Methods:**

Among all individuals diagnosed with type 1 diabetes between 1965 and 1979 in Finland (n = 5144) with at least one child with the same disease and a diabetes duration of 15 years or more, we compiled a population-based study cohort comprising 221 parent-offspring pairs. The original cohort was compiled by the Finnish Institute for Health and Welfare, and their offspring identified from the Finnish Central Population Register. The status of diabetic kidney disease and severe diabetic retinopathy (defined as retinal laser treatment) was determined by systematically reviewing the participants’ medical records. The follow-up ranged from the diabetes onset until 31 December 2020.

**Findings:**

In total, 56 (32%) parents developed severe albuminuria and 84 (51%) severe diabetic retinopathy during follow-up. Among the offspring of parents free of kidney disease, 23 (18%) developed moderate albuminuria, 10 (8%) developed severe albuminuria, and 1 (1%) developed kidney failure, while among offspring of parents with kidney disease, 20 (34%) developed moderate albuminuria, 10 (17%) developed severe albuminuria, and 1 (2%) developed kidney failure during follow-up. Among the offspring of parents free of severe diabetic retinopathy, 8 (9%) developed severe diabetic retinopathy, whereas the corresponding proportion was 29 (33%) among the offspring of parents with severe diabetic retinopathy. The presence of severe albuminuria in the parent increased the risk of offspring moderate albuminuria 2.27-fold (95% confidence interval 1.25–4.14, p = 0.0075) and offspring severe albuminuria 2.41-fold (1.00–5.83, p = 0.049), as compared to offspring of parents without kidney disease. The presence of kidney failure in the parent further increased the risk of offspring moderate albuminuria to 2.60-fold (1.33–5.07, p = 0.0051) and offspring severe albuminuria to 3.04-fold (1.21–7.65, p = 0.019). Severe diabetic retinopathy in the parent increased the risk of the same complication in the offspring 4.34-fold (1.98–9.50, p = 0.00024).

**Interpretation:**

Offspring of parents with diabetic kidney disease or severe diabetic retinopathy have a several-fold risk of developing the corresponding complication compared with offspring of parents without the complications. This observation highlights the importance of family history when assessing the complication risk among individuals with type 1 diabetes.

**Funding:**

10.13039/100015736Folkhälsan Research Foundation, Medical Society of Finland, 10.13039/100010113Wilhelm and Else Stockmann Foundation, 10.13039/501100013500Finnish Diabetes Research Foundation, 10.13039/501100016033Waldemar von Frenckell Foundation, Liv och Hälsa Society, 10.13039/501100002341Academy of Finland (grant number 316664), 10.13039/501100009708Novo Nordisk Foundation (grant number NNF OC0013659).

**Research in context**

**Evidence before this study**

We searched PubMed for publications in English until 1 September 2024 using the term “type 1 diabetes” and “familial clustering” in combination with “albuminuria”, “kidney disease”, “nephropathy”, or “retinopathy”. We also manually reviewed the reference lists of the identified publications to find additional eligible studies and uncovered that diabetic kidney disease and diabetic retinopathy tend to cluster among sibling pairs concordant for type 1 diabetes. However, the recurrence risk of these complications in parent-offspring pairs remains largely unknown, due to the limited number of individuals included in previous research.

**Added value of this study**

This population-based study with an extensive median follow-up of 19.5 years, reveals that diabetic kidney disease and diabetic retinopathy cluster among parent-offspring pairs with type 1 diabetes. Offspring have a 2.27-fold (95% confidence interval 1.25–4.14, p = 0.0075) higher risk of kidney disease if the parent has severe albuminuria, compared to those whose parents are free from manifest kidney disease. Additionally, severe diabetic retinopathy in the parent is linked to a 4.34-fold (1.98–9.50, p = 0.00024) increased risk of severe retinopathy in the offspring.

**Implications of all the available evidence**

Familial clustering of diabetic kidney disease and diabetic retinopathy occurs in type 1 diabetes, affecting both sibling pairs and parent-offspring pairs. This pattern is thought to be driven by a combination of genetic factors and shared environmental influences. The available evidence shows that family history offers a valuable insight into the risk of these diabetic microvascular co-morbidities and should, thus, not be overlooked.

## Introduction

Diabetic kidney disease is a severe microvascular complication that continues to affect approximately every third individual with type 1 diabetes, even with modern treatment strategies.^1^ It has been linked to an increased risk of cardiovascular disease and it is also the main contributing factor to the premature mortality associated with type 1 diabetes.^2^^,^^3^ Diabetic retinopathy, on the other hand, is a severe and potentially sight-threatening long-term complication of diabetes, also affecting the microvasculature. As a result of improved diabetes therapy, the incidence and prevalence of impaired vision due to diabetic retinopathy have decreased in Finland since the late 1990s.^4^ Despite this, it continues to be an important cause of visual impairment, particularly in the working-age population.^5^

Several environmental risk factors for diabetic kidney disease and diabetic retinopathy have been identified, with hyperglycemia being the most significant one. However, these risk factors cannot alone predict the development of diabetic kidney disease and diabetic retinopathy, indicating that genetic factors also play a role. Recent genetic studies have estimated that the heritability of diabetic kidney disease is 24–42% in type 1 diabetes and 6–33% for retinopathy in diabetes of any type.^6^^,^^7^

Studies have shown that familial clustering of diabetic kidney disease in type 1 diabetes does occur; however, these findings mainly stem from studies investigating siblings.^8^, ^9^, ^10^ In 1989, Seaquist et al. reported that 83% of siblings of kidney transplant recipients had albuminuria or kidney failure, compared to 17% of siblings of probands free of kidney disease manifestations. While the cohort was small, the study was still regarded as a landmark of its time.^11^ The arguably most comprehensive analysis in this field dates back to 2004. In this population-based analysis, Harjutsalo et al. showed that siblings of probands with overt albuminuria or kidney failure had a 2.3-fold risk to develop diabetic kidney disease compared with siblings of probands with moderate albuminuria or normal albumin excretion rate (AER).^12^

Less is known about the clustering of diabetic kidney disease among parent-offspring pairs with type 1 diabetes. As part of the Diabetes Control and Complications Trial (DCCT) in 1997, familial clustering of diabetic kidney disease was studied in a cross-sectional setting including both sibling pairs and parent-offspring pairs with type 1 diabetes.^13^ Relatives to a proband with diabetic kidney disease presented with an elevated risk of albuminuria, whereas the correlation of AER between a parent and their child with type 1 diabetes was not significant. All in all, reliable conclusions could not be drawn due to the limited number of study participants with manifest kidney disease. Besides this study, data drawn from parent-offspring pairs are scarce.

Familial clustering of diabetic retinopathy has also been observed, although even fewer studies have been conducted than for diabetic kidney disease. The results of the 1997 DCCT study found a significant correlation of the severity of diabetic retinopathy in parent-offspring pairs among 127 families.^13^ The familial clustering of diabetic retinopathy was also investigated in a sibling study conducted in the Finnish Diabetic Nephropathy (FinnDiane) cohort, in which the presence of proliferative retinopathy in the proband entailed an increased risk of retinopathy in the siblings of the proband with an odds ratio of 2.8.^14^ Similar results were attained by Monti et al.; however, the odds ratio in this study was markedly higher, namely 9.9.^10^

Taken together, diabetic kidney disease and diabetic retinopathy tend to aggregate within sibling pairs concordant for type 1 diabetes; yet, the familial clustering of these complications has not yet been fully explored. To obtain a broader understanding, we aimed to study whether the presence of diabetic kidney disease or severe diabetic retinopathy in the parent is associated with the offspring’s risk of these complications with a population-based, retrospective cohort study design.

## Methods

### Study population

The study was conducted using a study population previously established to investigate the parent-offspring recurrence risk of type 1 diabetes.^15^ The original population-based cohort was compiled by the Finnish Institute for Health and Welfare and consisted of all 5144 individuals diagnosed with type 1 diabetes below the age of 18 years in Finland between the years 1965 and 1979. The diabetes diagnosis of these individuals had been determined by their healthcare provider and was based on internationally recognized diagnostic criteria. Their offspring (n = 5291) were identified from the Finnish Central Population Register. The diabetes status in the offspring was defined until 2003, yielding 259 cases of type 1 diabetes. For this particular study, only offspring with a diabetes duration ≥15 years at study initiation were considered to ensure long enough diabetes duration to be able to reliably assess the incidence of diabetic co-morbidities. Consequently, 226 parent-offspring pairs met these criteria. After excluding cases of type 2 diabetes, HNF4A-MODY (MODY 1), HNF1A-MODY (MODY3), and diabetes of uncertain type, 221 parent-offspring pairs remained. The final study population consisted of 423 individuals in total: 221 offspring and 202 parents, whereof 184 parents with one child, 17 parents with two children, and one parent with three children with type 1 diabetes. The offspring included in the study were diagnosed with type 1 diabetes between 1982 and 2002.

### Kidney disease and retinopathy outcomes

In order to determine the study participants’ status of diabetic kidney disease and diabetic retinopathy, we systematically reviewed the participants’ medical records and laboratory test results. Information about the study participants’ place of residency was obtained from the Finnish Population Information System. Medical records and laboratory test results from the diabetes onset up until 31 December 2020 were ordered from hospitals and public health care centers in these areas.

The diabetic kidney disease status of the participants was determined by assessing urinary albumin-to-creatinine ratio (ACR) from spot urine or AER values based on timed overnight or 24-h urine collections. In all, 39% of the offspring participants’ diagnostic albuminuria measurements were ACRs (mg/mmol), 55% were overnight AERs (μg/min), and 5% were 24-h AERs (mg/24 h). In contrast, 12% of the parent participants’ diagnostic albuminuria measurements were ACRs (mg/mmol), 45% were overnight AERs (μg/min), and 22% were 24-h AERs (mg/24 h). In the absence of albuminuria determinations, a protein excretion rate of >0.5 g/24 h was considered diagnostic for severe albuminuria (1% and 21% of the diagnostic urinary determinations in the offspring and parents, respectively). Diabetic kidney disease was categorized into four categories: normal AER, moderate albuminuria, severe albuminuria, and kidney failure. Normal AER was defined as ACR < 3 mg/mmol, AER < 20 μg/min, and AER < 30 mg/24 h. Moderate albuminuria was defined as ACR 3–30 mg/mmol, AER 20–200 μg/min, or AER 30–300 mg/24 h in two out of three subsequent albuminuria measurements. As more precise methods for determining albuminuria were not developed until the early 1980s, moderate albuminuria was only assessed for the offspring participants. Severe albuminuria was defined as ACR > 30 mg/mmol, AER > 200 μg/min, or AER > 300 mg/24 h in two out of three subsequent albuminuria measurements. Kidney failure was defined as initiated kidney replacement therapy, that is, dialysis or kidney transplantation.

In line with the kidney disease outcomes, the retinopathy status was also determined based on the participants’ medical records. The outcome of interest, severe diabetic retinopathy, was defined as a history of retinal laser treatment. We considered the time point for the first-ever laser treatment.

With respect to diabetic kidney disease, follow-up data were missing due to unattended albuminuria screenings or unavailable medical records in 33 (15%) parent-offspring pairs (data missing either for parent, offspring, or both). The number of parent-offspring pairs with missing data for severe diabetic retinopathy was 42 (19%). The excluded and included individuals did not differ in terms of age at diabetes onset, sex distribution, or the proportion of parents with multiple children included in the study (*data not shown*).

### Ethics

The study was approved by the Finnish Institute for Health and Welfare (approval number THL/786/6.02.00/2016) and Statistics Finland (approval number TK53-26-16). Informed consent was not required due to the register-based study design.

### Statistics

All statistical analyses were performed using the R open-source software (http://www.r-project.org) version 3.6.1. Cumulative incidence curves for the outcomes of interest (diabetic kidney disease and severe diabetic retinopathy in offspring) were generated using the Kaplan–Meier estimator, with parental status of the outcomes as stratification. The origin and start time for the survival analysis was defined as the offspring’s diabetes diagnosis, and the follow-up ended at outcome event, death, emigration or loss to follow-up due to other causes, or 31 December 2020—whichever occurred first. The log-rank test was used to assess differences between groups. Cox proportional hazards regression was used to analyze the association between the parents’ diabetic kidney disease and severe diabetic retinopathy status, and the risk of diabetic kidney disease or retinopathy outcomes in the offspring. Multivariable Cox regression analyses adjusted for offspring sex and offspring age at diabetes onset were also performed. Due to the nonlinear relationship between offspring age at diabetes onset and offspring diabetic kidney disease/severe diabetic retinopathy risk (p for nonlinearity < 0.05), we included age at onset as a nonlinear term using restricted cubic splines with three knots. The number of knots was selected based on Akaike information criterion minimization and the knots placed at the 10th, 50th, and 90th percentile, corresponding to 2.1, 6.8, and 14.4 years, respectively. The proportional hazards assumption was met for all regression models, as assessed using Schoenfeld residuals. To evaluate the potential modifying effect of the sex of the parent on the association between the exposure and outcome variables of interest, we studied interactions both on a multiplicative and an additive scale and assessed relative excess risk due to interaction (RERI) -values.^16^ The results are presented as hazard ratios with 95% confidence intervals. p values < 0.05 were considered statistically significant.

### Role of funding source

The funders had no role in the design of the study, data collection, data analysis, data interpretation, writing of the manuscript, or the decision to submit the manuscript for publiction.

## Results

The attained follow-up times for the offspring were 4061 person years for moderate albuminuria (median follow-up time 19.1 years, IQR 16.1–23.1), 4403 person years for severe albuminuria (median 19.5 years, IQR 17.1–23.7), and 4682 person years for kidney failure (median 20.6 years, IQR 18.1–25.6). Correspondingly for the parents, the attained follow-up times were 6935 person years for severe albuminuria (median follow-up time 41.8 years, IQR 32.0–46.7) and 8176 person years for kidney failure (median 43.9 years, IQR 38.1–47.9). Regarding severe diabetic retinopathy, the attained follow-up times were 4162 person-years for the offspring (median 19.3 years, IQR 17.3–23.0) and 6240 for the parents (median 38.1 years, IQR 20.8–43.2).

On average, the offspring were diagnosed with type 1 diabetes at a younger age than the parents. The median age at diabetes onset for the offspring was 6.8 years (IQR 3.6–10.7 years), whereas the median age at onset for the parents was 11.9 years (IQR 9.1–14.3 years). In the offspring group, 112 (51%) of the study participants were male and 109 (49%) female. Among the parents, 134 (66%) of the parents were male and 68 (34%) female. The clinical characteristics available for comparison between the groups with and without diabetic kidney disease and severe diabetic retinopathy are presented in Table 1.

**Table 1:** Baseline clinical characteristics, stratified by the presence of diabetic kidney disease and severe diabetic retinopathy, presented separately for parents and offspring.

Parents			
	Free of diabetic kidney disease	Presence of diabetic kidney disease	p value
Age at diabetes onset (years)	12.6 (9.5–14.4)	11.2 (7.7–13.8)	<0.0001
Sex (female)	48 (40.7%)	13 (22.8%)	0.031
	Free of severe diabetic retinopathy	Presence of severe diabetic retinopathy	p value

Age at diabetes onset (years)	13.1 (9.8–14.5)	11.3 (8.3–14.1)	<0.0001
Sex (female)	36 (43.3%)	22 (25.6%)	0.023

Offspring			
	Free of diabetic kidney disease	Presence of diabetic kidney disease	p value
Age at diabetes onset (years)	7.0 (4.0–10.7)	8.7 (3.4–11.4)	<0.0001
Sex (female)	92 (47.7%)	14 (60.9%)	0.33
	Free of severe diabetic retinopathy	Presence of severe diabetic retinopathy	p value

Age at diabetes onset (years)	6.8 (3.8–11.0)	6.5 (3.6–9.2)	<0.0001
Sex (female)	79 (47.9%)	25 (56.8%)	0.38

Kidney disease was defined as a diagnosis of severe albuminuria. Data are presented as median (IQR) for age at diabetes onset and n (%) for sex. p values denote between-group differences.

**Fig. 1:**
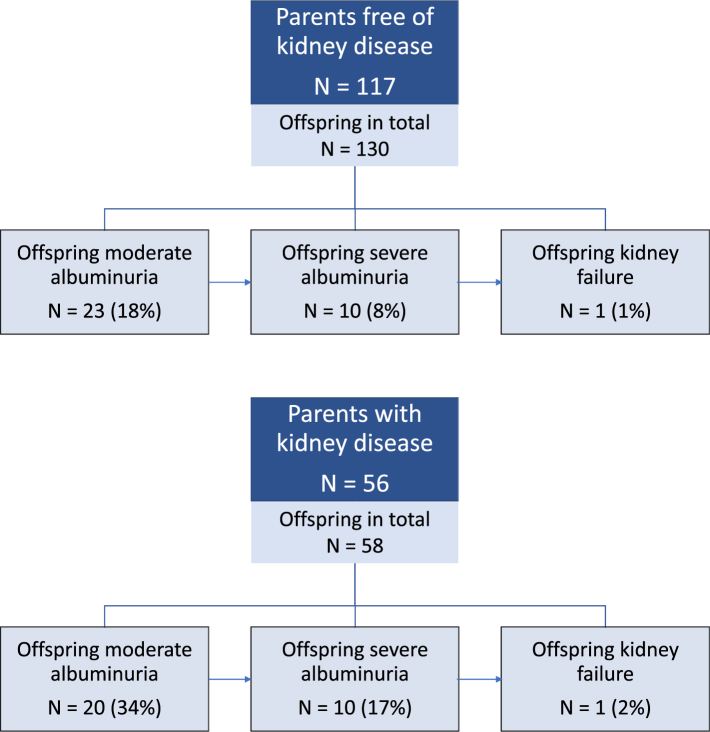
**Flow chart depicting the status of diabetic kidney disease among the offspring, stratified by kidney disease status in the parent.** Kidney disease in the parents was defined as a diagnosis of severe albuminuria.

**Fig. 2:**
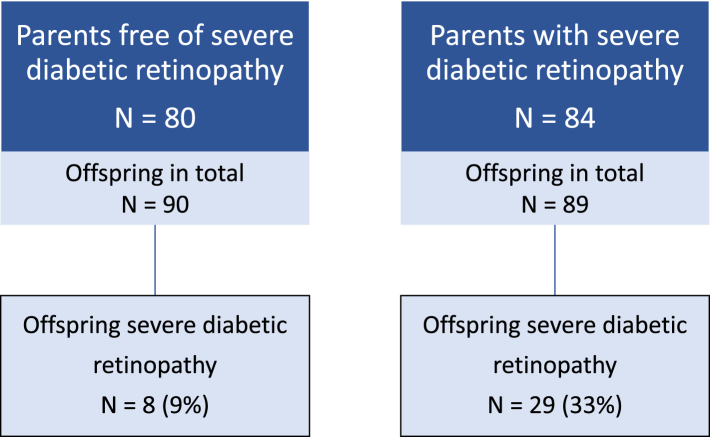
**Flow chart depicting the status of severe diabetic retinopathy among the offspring, stratified by retinopathy status in the parent.** Severe diabetic retinopathy was defined as a history of laser treatment.

Out of the 173 parents included in the kidney disease analyses, 56 (32%) developed severe albuminuria and 26 (15%) developed kidney failure during the follow-up period. Correspondingly, 84 (51%) of the 164 parents included in the retinopathy analyses developed severe diabetic retinopathy during follow-up. As shown in Fig. 1, the occurrence of kidney disease outcomes was markedly higher among the offspring of parents with kidney disease compared to the offspring of parents free of kidney disease (Chi-squared test for between-group difference p = 0.019, incorporating any severity of kidney disease among offspring). A similar phenomenon was noted in the incidence pattern of severe diabetic retinopathy among the offspring (p = 0.00019 with the Chi-squared test; rates portrayed in Fig. 2).

### Diabetic kidney disease

We analyzed male and female participants together since no evidence of interaction between the parents’ sex and the kidney outcome events studied was observed in multiplicative interaction analyses (p for interaction 0.11). The interaction analyses were also revisited from an additive perspective for each exposure-outcome pair separately, as is reported below.

**Fig. 3:**
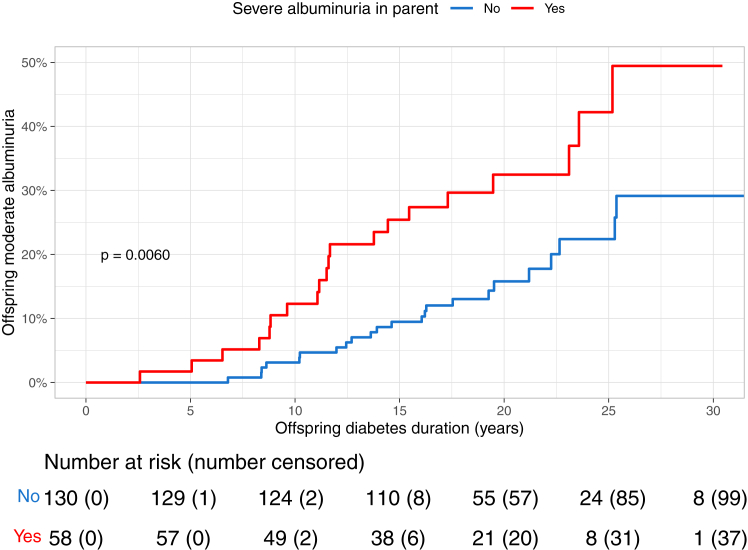
**The cumulative incidence of moderate albuminuria in the offspring stratified by the parents’ status of severe albuminuria during follow-up.** Log-rank p denotes the between-group difference.

The offspring of the parents who had developed severe albuminuria during follow-up had an increased risk to develop moderate albuminuria in comparison to the offspring of the parents free of kidney disease, HR 2.27 (95% CI 1.25–4.14), p = 0.0075. No evidence of additive interaction between parental sex and severe albuminuria for the risk of offspring moderate albuminuria was seen (RERI = 0.82 [95% CI -1.01 to +2.65]). Kidney failure in the parent further increased the risk of moderate albuminuria in the offspring, HR 2.60 (95% CI 1.33–5.07), p = 0.0051. Likewise, no evidence of interaction between parental sex and kidney failure status was seen (RERI = 0.90 [95% CI -2.03 to 3.84]). The Kaplan–Meier incidence curves for moderate albuminuria in the offspring stratified by the parents’ status of severe albuminuria started to diverge early (Fig. 3). The 15-year cumulative incidences for moderate albuminuria were 9.5% (95% CI 4.2–14.4) in the offspring whose parents were free of kidney disease, and 25.4% (95% CI 12.9–36.1) in the offspring whose parents had developed kidney disease. The corresponding 25-year cumulative incidences for moderate albuminuria were 22.4% (95% CI 12.2–31.4) in those offspring whose parents were free of kidney disease and 42.2% (95% CI 22.7–56.8) in those whose parents had developed kidney disease.

Additionally, the offspring’s risk of severe albuminuria was increased in the group in which the parents had severe albuminuria, compared to the group with parents free of kidney disease, HR 2.41 (95% CI 1.00–5.83), p = 0.049. No evidence of additional interaction was found between parental sex and severe albuminuria in relation to offspring severe albuminuria risk (RERI = 1.02 [95% CI -2.33 to 4.36]). The presence of kidney failure in the parent further heightened the risk to 3.04-fold (95% CI 1.21–7.65), p = 0.019. The same was observed for the interaction between parental sex and kidney failure (RERI = 2.62 [95% CI -2.24 to 7.48]). The 15-year cumulative incidence for offspring’s severe albuminuria was 1.6% (95% CI 0.0–3.7) in those with parents free of kidney disease and 7.5% (95% CI 0.2–14.3) in those with parents with kidney disease. The corresponding 25-year cumulative incidences were 11.8% (95% CI 2.5–20.3) for those with parents free of kidney disease and 16.2% (95% CI 5.2–26.0) for those with parents with kidney disease.

The association between offspring albuminuria and parental kidney disease status persisted even when adjusting for offspring sex and offspring age at diabetes onset in a multivariable Cox regression analysis: the adjusted HR for offspring moderate albuminuria was 2.32 (95% CI 1.26–4.27), p = 0.0070 and offspring severe albuminuria 2.66 (95% CI 1.09–6.50), p = 0.032.

The main results attained from Cox regression analyses regarding diabetic kidney disease are summarized in Table 2.

**Table 2:** Summary of Cox regression analysis results regarding diabetic kidney disease and severe diabetic retinopathy.

Diabetic kidney disease	HR	HR (95% CI)	p value
	Parents free of kidney disease	Parents with diabetic kidney disease	
Offspring moderate albuminuria	1 (ref)	2.27 (1.25–4.14)	0.0075
Adjusted	1 (ref)	2.32 (1.26–4.27)	0.0070
Offspring severe albuminuria	1 (ref)	2.41 (1.00–5.83)	0.049
Adjusted	1 (ref)	2.66 (1.09–6.50)	0.032
**Severe diabetic retinopathy**	**Parents free of severe diabetic retinopathy**	**Parents with severe diabetic retinopathy**	

Offspring severe diabetic retinopathy	1 (ref)	4.34 (1.98–9.50)	0.00024
Adjusted	1 (ref)	4.79 (2.18–10.52)	<0.0001

HR, hazard ratio; CI, confidence interval; ref, reference. Multivariable analyses were adjusted for offspring sex and age at diabetes onset. Regarding age at onset, nonlinearity was allowed using restricted cubic splines with three knots. Parental kidney disease was defined as a diagnosis of severe albuminuria. p values denote between-group differences.

### Diabetic retinopathy

**Fig. 4:**
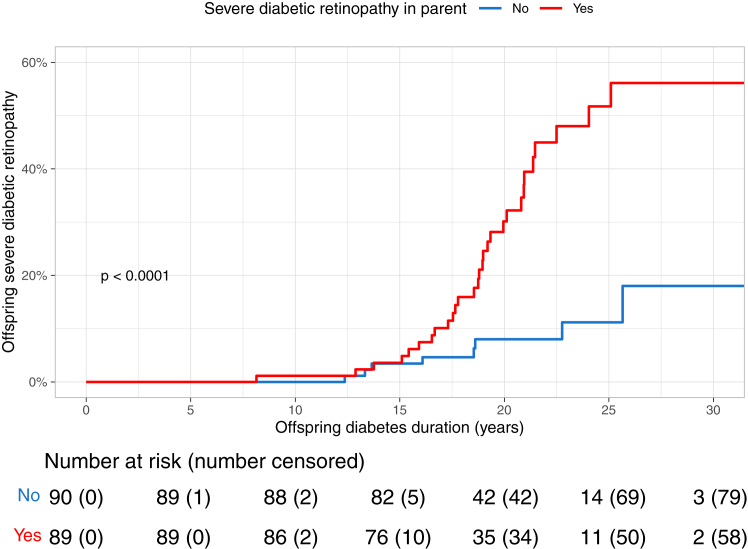
**The cumulative incidence of severe diabetic retinopathy in the offspring stratified by the parents’ status of severe diabetic retinopathy during follow-up.** Log-rank p denotes the between-group difference.

We analyzed male and female participants together since no evidence of interaction in a multiplicative model between the parents’ sex and the retinopathy outcome studied was observed (p for interaction = 0.24). The offspring’s risk of severe diabetic retinopathy was significantly increased if the parent had severe diabetic retinopathy, HR 4.34 (95% CI 1.98–9.50), p = 0.00024. When adjusting for offspring sex and offspring age at diabetes onset the link persisted, HR 4.79 (95% CI 2.18–10.52), p < 0.0001. RERI for interaction between parental sex and severe diabetic retinopathy was 1.36 (95% CI -4.84 to 7.56) indicating no significant departure from additivity. The Kaplan–Meier incidence curves for severe diabetic retinopathy in the offspring stratified by the parents’ status of severe diabetic retinopathy are shown in Fig. 4. The 15-year cumulative incidence for severe diabetic retinopathy in the offspring were similar in both groups: 3.4% (95% CI 0.0–7.2) in the group in which the parents did not have severe diabetic retinopathy, and 3.6% (95% CI 0.0–7.5) in the group in which the parents had developed severe diabetic retinopathy. However, after this, the cumulative incidence started to diverge, as is illustrated in Fig. 4. The 25-year cumulative incidence for severe diabetic retinopathy in the offspring were 11.2% (95% CI 2.1–19.4) in the group in which the parents did not have severe diabetic retinopathy and 51.7% (95% CI 34.1–64.7) in the group in which the parents had developed severe diabetic retinopathy.

The main results attained from Cox regression analyses regarding severe diabetic retinopathy are summarized in Table 2.

## Discussion

This population-based, retrospective cohort study shows that diabetic kidney disease clusters in families with type 1 diabetes. Notably, to the best of our knowledge, this is the first time such a conclusion can be drawn by specifically analyzing parent-offspring pairs. In our study, offspring of parents with severe albuminuria had a 2.3-fold risk of diabetic kidney disease, compared with offspring of parents without kidney disease. It is noteworthy that the presence of kidney failure (that is, the need for kidney replacement therapy—the most advanced stage of kidney disease) in the parent further increased the offspring recurrence risk of albuminuria. Moreover, we demonstrated that not only diabetic kidney disease, but also diabetic retinopathy, shows a higher incidence among the offspring of parents affected with the same complication. More specifically, severe diabetic retinopathy in the parent was associated with a 4.3-fold increased risk of severe diabetic retinopathy in the offspring. Based on the results of this observational study, we cannot determine whether the increased recurrence risk of these diabetic complications derives from genetic factors or shared environmental factors in the families, but it is likely that both play a role.

Familial clustering of diabetic kidney disease and diabetic retinopathy has been reported in a number of studies analyzing sibling pairs concordant for type 1 diabetes.^8^, ^9^, ^10^, ^11^, ^12^, ^13^, ^14^ Interestingly, our results were strikingly similar to the results in a sibling study by Harjutsalo et al. in 2004,^12^ in which the risk of diabetic kidney disease in type 1 diabetes was 2.3-fold in siblings of probands with advanced diabetic kidney disease compared with siblings of probands free of kidney disease. Notably, these two studies on the familial clustering of diabetic kidney disease originate from a common cohort of 5144 probands diagnosed with type 1 diabetes between 1965 and 1979. Previous research within this cohort has investigated clustering of type 1 diabetes in siblings and also diabetic kidney disease in these sibling pairs, as well as transmission of type 1 diabetes from parents to offspring.^12^^,^^15^^,^^17^ The current study extends the investigation of familial clustering within the cohort to diabetic kidney disease among parent-offspring pairs with type 1 diabetes. The similarity between the results of the current study and the previous sibling study aligns with the assumption that the sibling risk for unlinked loci in multifactorial traits is always at least as large as the offspring risk.^18^

The lack of previous studies on the familial clustering of diabetic complications among parent-offspring pairs most likely arises from the inherent challenge of obtaining an adequately representative population to explore the phenomenon. The reasons behind this challenge are multifactorial. First, despite its continuously rising burden, the prevalence of type 1 diabetes remains relatively low on a global scale. For instance, in 2021, the estimated global prevalence of type 2 diabetes was 537 million *versus* 8.4 million for type 1 diabetes at the corresponding time point.^19^^,^^20^ Second, over 80% of cases of type 1 diabetes are sporadic, meaning that they occur in individuals without a known family history of diabetes at the time of the diagnosis.^21^^,^^22^ Further adding to this aspect, it has been reported from the same initial population as ours (all individuals diagnosed with type 1 diabetes in Finland between 1965 and 1979) that both female and male individuals with diabetes have a smaller number of live births compared to the diabetes-free background population.^23^ Parent-offspring pairs in which both parties are afflicted with type 1 diabetes are, thus, rather rare. Third, in order to explore the offspring recurrence risk of microvascular complications, a substantial proportion of both the parents and the offspring must have developed the disease of interest–in this case diabetic kidney disease or diabetic retinopathy. We have previously shown that the incidence of diabetic kidney disease has declined over time, with the 25-year cumulative incidence of severe albuminuria standing at only 11–12% after 25 years of diabetes among those with diabetes onset in 1980–1999.^1^ The falling temporal trend reportedly also applies to the incidence of severe diabetic retinopathy in type 1 diabetes.^24^ While the declining burden of these diabetic complications is undoubtedly a desired outcome, it poses challenges to study designs such as ours.

To tackle these obstacles regarding the study population, our initial cohort was a population-based sample comprising all individuals diagnosed with type 1 diabetes between 1965 and 1979 below the age of 18 years (n = 5144), drawn from the country with the all-time highest incidence of type 1 diabetes globally.^25^ All children of these participants with type 1 diabetes and a diabetes duration of at least 15 years were included in the study (n = 221), and both the diabetes as well as the kidney disease/diabetic retinopathy status was carefully scrutinized by reviewing medical records. The follow-up time was vast, ranging up until the end of 2020—that is, up to 55 years for the study participants with the earliest diabetes onset. Despite this careful selection and deliberate outcome characterization, cases of diabetic kidney disease and severe diabetic retinopathy within our sample were sparse. Nonetheless—to the best of our knowledge—our study represents the most comprehensive model to date for examining the recurrence rate of diabetic kidney disease as well as retinopathy among offspring of individuals with type 1 diabetes. Therefore, the insights we present contribute a novel perspective to the field.

The extensive follow-up period naturally raises the possibility that changes in diabetes practice guidelines over the long timeframe (1965–2020) may have influenced both the risk and expression of the studied complications, potentially affecting the results. This hypothesis would align with our previous findings, indicating a decline in the cumulative incidence of albuminuria among individuals diagnosed with type 1 diabetes in the 1980s compared to earlier disease onset—likely due to the introduction of ACE inhibitors and their kidney-protective benefits.^1^ While this is a limitation and should be considered when interpreting the results, the lengthy follow-up period and real-world context also stand out as key strengths of the study.

The etiology of diabetic kidney disease is complex and is influenced by genetic, epigenetic, and environmental factors. We noticed that the HR for the parent-offspring risk was higher for the offspring having severe albuminuria (HR 2.41, 95% CI 1.00–5.83) than for having moderate albuminuria (HR 2.27, 95% CI 1.25–4.14), and that parental kidney failure further increased the risk of both outcomes. These findings are in line with the heritability estimates obtained from genome-wide genotyping data (of unrelated individuals) indicating higher heritability for more severe forms of diabetic kidney disease.^26^ Novel findings in genetic research have broadened our understanding of the etiology of diabetic kidney disease and, to date, nearly 80 genetic risk loci for diabetic kidney disease have been identified. However, the updated genetic knowledge has not yet translated into clinical practice.^6^

Besides genetically and epigenetically inherited traits, environmental and lifestyle influences shared within families can also mimic the pattern of genetic transmission. To the best of our knowledge, no studies have specifically investigated how shared environmental factors impact the recurrence rate of diabetic kidney disease and other diabetic long-term complications between family members concordant for type 1 diabetes. However, several factors commonly shared within families, often further evolving into lifelong habits after childhood, have been linked to an increased risk. For instance, physical activity and exercise habits have been highlighted as risk factors for the development and progression of albuminuria in several study settings.^27^ Furthermore, given that the parents and offspring were concordant for type 1 diabetes in our cohort, adherence to treatment may also play a role, as offspring could adopt management patterns learned from their parents. Other potential influences, though more speculative, include pathogen exposure and pregnancy conditions, particularly among offspring to mothers with type 1 diabetes. All in all, it is challenging to evaluate the relative influence of genetic factors and environmental factors associated with the shared environment in families for the development of diabetic kidney disease and other diabetic complications. Traditional heritability studies, thus, continue to be an important tool for considering the combined influence of the genes and the environment.

Recent studies have estimated the heritability of diabetic retinopathy to be 6–33%; yet, the genetics behind diabetic retinopathy in type 1 diabetes remains substantially understudied.^7^^,^^28^ Our study showed that a family history of diabetic retinopathy entailed a markedly increased risk of the complication. Up until 15 years of follow-up, there was no between-group differences in the cumulative incidence of severe diabetic retinopathy among offspring of parents with and without severe diabetic retinopathy, attributable to the overall low occurrence of laser treatment-requiring retinopathy this early in the course of diabetes. However, after this time point, the cumulative incidence curves started to diverge notably. At 25 years of follow-up, the cumulative incidence was only 11% for the offspring of parents free of severe diabetic retinopathy, whilst being as high as 52% for the offspring of parents with severe diabetic retinopathy. In other words, the difference was of even greater magnitude than that observed for albuminuria. These striking results further underline the importance of assessing family history of disease, in the context of diabetic retinopathy risk as well.

Offspring of female and male parents were analyzed together since no interaction between the parents’ sex and the outcome events studied was observed. This remark applied across all explored outcomes (offspring moderate albuminuria, severe albuminuria, and severe diabetic retinopathy) as well as all exposure variables (parental severe albuminuria, kidney failure, and severe diabetic retinopathy). This is in contrast to some previous studies indicating that the sex of the parent may affect the offspring risk of type 1 diabetes and its complications. For instance, in the FinnDiane cohort, maternal type 1 diabetes was associated with higher odds of kidney disease in the offspring with type 1 diabetes, whereas paternal type 1 diabetes was not.^29^ The reasons underlying the discrepancy between the FinnDiane Study and ours are not obvious; yet, the FinnDiane Study lacked data on the kidney disease status of the parent with diabetes, potentially accounting for the observed difference. Furthermore, offspring of fathers with type 1 diabetes have been shown to express a higher recurrence risk of type 1 diabetes than offspring of mothers with type 1 diabetes.^15^^,^^30^ Interestingly, the distribution of male and female parents in this study was concordant with this inheritance pattern, as 66% of the parents were male and only 34% female, although the sex distribution was more even in the cohort from which these probands were originally drawn (55% male and 45% female).

Family health history, defined as positive when the relative risk exceeds two,^31^ plays a crucial role in assessing an individual's disease risk. All HRs in our study were greater than two, although due to the reasons related to study population size, the confidence intervals are wide. Family health history has been found to be one of the strongest predictors of disease risk, even in the era of genome-wide association studies and polygenic risk scores.^31^ Therefore, our findings are clinically important. Family history of type 1 diabetes is frequently asked when diagnosing type 1 diabetes, but even more critical might be assessing the history of diabetic complications of the affected family members. A positive history could prompt earlier preventive measures, such as enhanced screenings, timely interventions, and lifestyle modifications.

However, there are some factors to consider regarding the generalizability of heritability studies, such as ours. The heritability of a disease is influenced by certain population-specific factors, including allele frequencies, the impact of gene variants, and variation due to environmental factors.^32^ Some demographic factors in Finland may further play a role in the external validity of our results, such as the characteristic genetic homogeneity, as well as a particularly high incidence of type 1 diabetes on the global scale. All in all, the magnitudes of our observations do not necessarily align with what would be observed in other populations if assessed. However, given the consistent discovery of increased offspring recurrence risks for both diabetic kidney disease and retinopathy in our population, it is reasonable to assume familial clustering of these microvascular complications in parent-offspring pairs in other populations as well.

An important limitation of our study that needs to be acknowledged is the possibility of selection bias due to complete-case analysis, due to the exclusion of individuals with unattended albuminuria screenings or unavailable medical records. Another is possibility of selection bias due to the inherent structures of hazard ratios.^33^ Additionally, our study had limited access to clinical measures such as glucose balance and socio-demographic factors, which could have been valuable as confounders in the multivariable regression models. Consequently, this may also have led to an overestimation of the HRs.

Furthermore, some potential limitations with respect to the explored outcomes need to be addressed. First, reliable conclusions regarding offspring kidney failure could not be drawn since only two of the offspring study participants developed kidney failure during follow-up. Second, a comprehensive view including all microvascular diabetes complications could not be presented since we were unable to obtain data regarding the participants’ status of diabetic neuropathy. Third, different assessment methods for albuminuria were used since the information was obtained from real-world data over a long time-period with changes in practice. Nevertheless, agreement between the different assessments methods is high,^34^ and additionally, the diagnostic thresholds to define moderate and severe albuminuria in the study are concordant with international guidelines—irrespective of the assessment method.

In conclusion, these findings show the occurrence of familial clustering of diabetic kidney disease and diabetic retinopathy in type 1 diabetes through a unique, population-based study of parent-offspring pairs. Over a vast follow-up period of more than 50 years, we observed a several-fold increased risk of these complications in offspring of parents with the same condition. These findings underscore that family history can tell a lot about the risk of different diseases and should not be overlooked when assessing the risk of diabetic kidney disease and diabetic retinopathy—even amidst the advancements in the current *omics* era.

## Contributors

IH, FJS, and VH designed the study, acquired the data, and did the statistical analyses. IH, FJS, and VH also had access to and verified the underlying data. All authors interpreted the results. IH and FJS wrote the manuscript, which was critically reviewed by NS, LMT, P-HG, and VH. All authors read and approved the final version of the manuscript. VH had final responsibility for the decision to submit for publication.

## Data sharing statement

The study data will not be available because the EU General Data Protection Regulation does not allow the distribution of patient-level data.

## Declaration of interests

FJS reports receiving lecture fees from AstraZeneca and Boehringer Ingelheim. P-HG reports receiving lecture fees from Astellas Pharma, AstraZeneca, Bayer, Berlin Chemie, Boehringer Ingelheim, Eli Lilly, Elo Water, Genzyme, Medscape, Menarini, Merck, Sharp & Dohme, Mundipharma, Novartis, Novo Nordisk, PeerVoice, Sanofi, and Sciarc. P-HG reports being an advisory board member for AbbVie, Astellas Pharma, AstraZeneca, Bayer, Boehringer Ingelheim, Eli Lilly, Janssen Pharmaceuticals, Medscape, Merck, Sharp & Dohme, Mundipharma, Nestlé, Novartis, Novo Nordisk, and Sanofi. None of these entities participated in the design or interpretation of the study. IH, NS, LMT, and VH declare no competing interests.
